# Active legumain promotes invasion and migration of neuroblastoma by regulating epithelial-mesenchymal transition

**DOI:** 10.1515/biol-2022-0012

**Published:** 2022-06-21

**Authors:** Min Zhang, Jianhua Zhu, Wei Wang, Zhiteng Jiang

**Affiliations:** Department of Emergency & Trauma Surgery, Shanghai University of Medicine and Health Sciences, Affiliated Zhoupu Hospital, Shanghai 201318, P. R. China; Department of Pediatric Surgery, Xinhua Hospital, School of Medicine, Shanghai Jiao Tong University, Shanghai 200092, P. R. China; Colloge of Pharmacy, Shanghai University of Medicine and Health Sciences, Shanghai 201318, P. R. China

**Keywords:** legumain, asparagine endopeptidase, neuroblastoma, epithelial-mesenchymal transition, metastasis

## Abstract

Neuroblastoma (NB) is a commonly occurring malignancy in children. Epithelial-mesenchymal transition (EMT) is an adaptive change in promoting tumor metastasis. As an important factor in regulating tumor metastasis, whether legumain could promote metastasis of NB by EMT is still unexplored. Legumain is the active form of prolegumain, abundant in tumor plasma. So in the current study, different forms of legumain were identified in NB. Second, correlation analysis of N-cadherin and active legumain was identified by western blot analysis. Third, legumain gene amplification or gene knockdown were proceeded to examine the effect of legumain on EMT by scratch and transwell assay; meanwhile, active mature legumain or its asparagine endopeptidase (AEP) inhibitor was also added in. Finally, legumain can be detected differently in NB cells. Changes in legumain could influence NB metastasis by regulating EMT markers (e.g., N-cadherin, vimentin, and slug). Besides, the effect of legumain on EMT by its AEP activity was proved by intervention experiment of AEP gene transfection and gene knockdown experiments or adding recombinant human legumain suspension or specific inhibitor of AEP in NB cells (*p* < 0.05). These results suggest that legumain can promote invasion and migration of NB by regulating EMT, and EMT of NB is regulated by AEP activity of legumain, which can be inhibited by a specific AEP inhibitor.

## 1 Introduction

Neuroblastoma (NB), a solid tumor caused by rapid division of undifferentiated neuroblasts, is the most common malignancy affecting children aged <5 years, with an incidence rate of 0.3/100,000–5.5/100,000 [1,2], accounting for 10% of pediatric malignancies and 15% of children’s deaths from tumors [3]. Approximately 650 new cases of NB are diagnosed in the USA every year, 90% of patients are diagnosed before 5 years of age, and 70% of patients have distal tumor metastasis while being diagnosed.

Adaptive changes in the tumor microenvironment and cells promote tumor migration and metastasis. A large number of processes influence the tumor metastasis and migration process, such as chemotaxis [4], microRNA [5], long non-coding RNAs [6], and also proteases.

Proteases present in the tumor microenvironment and within tumor cells can regulate tumor invasion and migration by regulating factors in the tumor cytoplasm and tumor stroma [7]. Legumain is a conservative cysteine protease belonging to the C13 family that is overexpressed in solid tumors but is scarce in normal tissues [8]. It can be presented with three different forms: prolegumain, asparagine carboxypeptidase (ACP), and asparagine endopeptidase (AEP, also called legumain) under different atmospheres. Correlation analysis in solid tumors, such as gastric tumors [9], colon cancer [10], cervical cancer [11], ovarian tumors [12], and breast cancer [13], showed that the overexpression of legumain is associated with a poor prognosis. Legumain is highly abundant in tumor stroma and is also found in the cytoplasm and on the surface of tumor cells. It is abundant in M2 macrophages (tumor-associated macrophages) [14] and tumor blood vessels that facilitate tumor metastasis and migration. It promotes tumor metastasis mainly by hydrolyzing metastasis-related substrates in the tumor stroma, for example, fibronectin [15], progelatinase A [16], cathepsin L [17], and matrix metalloproteinases (MMPs). In addition, it can act as a different type of protease in other situations [18]and also as a transcription factor in the nucleus of colon cancer cells [19].

Epithelial-mesenchymal transition (EMT) is an important process that allows tumor cells to change, escape, and migrate to distant sites [20,21]. As cells undergo EMT, they also develop a mesenchymal phenotype and start expressing mesenchymal markers such as N-cadherin and vimentin [22]. In a study based on a rapid autopsy program for patients with pancreatic cancer, 75% of primary tumors with mesenchymal features developed metastatic lesions in the liver and lung [23].

Legumain plays an important role in regulating metastasis-related factors in the tumor stroma, but it is unclear whether it plays a role in tumor metastasis-related EMT in NB. The purpose of this investigation was to determine whether legumain is involved in EMT of NB, and if so, through what mechanism. Plasmid transfection to produce legumain gene overexpression in SK-N-BE2 cells and gene knockdown by siRNA in SH-SY5Y and IMR32 cells were used to evaluate the legumain effect on the EMT of NB. In addition, whether its effect on EMT was based on its AEP activity was evaluated using activated legumain or specific legumain inhibitor experiments in NB.

## 2 Materials and methods

### 2.1 Legumain expression in NB cell lines and NB tumor tissues

The human NB cell lines SH-SY5Y, IMR32, and SK-N-BE2 were purchased from the Type Culture Collection of the Chinese Academy of Sciences, Shanghai, China. These three cell lines were, respectively, grown in DMEM/F12, MEM/EBSS, and RPMI 1640 (Gibco; Thermo Fisher Scientific, Inc., Waltham, MA, USA) culture medium containing 10% fetal bovine serum (FBS; Gibco; Thermo Fisher Scientific, Inc.) at 37°C in a humidified 5% CO_2_ atmosphere. Legumain expression of the three cell lines was assayed by western blot analysis. Adherent cells were resuspended in RIPA buffer mixed with a protease inhibitor cocktail (1:1,000; Thermo Fisher Scientific, Inc.) and then centrifuged at 10,000*g* for 15 min at 4°C. The protein supernatant was transferred to new tubes and qualified by a BCA kit. Equal amounts of protein samples (10 µg/well) were electrophoretized by 10% SDS-PAGE electrophoresis and transferred to polyvinylidene fluoride（PVDF) membranes (Roche; Mannheim, Germany). After blocking with 5% skimmed milk, incubated with a primary antibody against legumain (1:500; cat. no. ab125286, Abcam, Cambridge, MA, USA) overnight and secondary antibody for 1 h, the membrane was incubated with SuperSignal West Pico (Thermo Fisher Scientific, Inc.) and then exposed.

The location of legumain in tumor cells and solid tumor tissues was examined by immunofluorescence assays. Glass slides with attached SH-SY5Ycells were fixed in formaldehyde for at least 30 min, washed twice with phosphate-buffered saline (PBS), blocked with 5% FBS for 2 h, and incubated with a primary antibody against legumain (1:500; cat. no. ab125286) overnight at 4°C and then with a secondary antibody against anti-rabbit IgG fluorescein (1:500; cat. no. ab150077) for 1 h in the dark at 37°C. The cell nuclei were stained with DAPI (10 mg/mL, Beyotime, SHH, CHN) for 30 min, then mounted with coverslips and observed by fluorescence microscope. Paraffin-embedded tissue sections of a tumor-bearing mouse model of SH-SY5Y, which was deparaffinized, hydrated, and incubated first with a primary antibody against legumain at 4°C overnight, were performed and then proceeded with the procedures as above.

### 2.2 N-Cadherin and legumain expression in NB tumor samples

Tumor samples from nine cases of NB were collected in Shanghai Xinhua Hospital. Samples were pulverized and resuspended in RIPA buffer with a protease inhibitor cocktail and then centrifuged at 10,000*g* for 15 min at 4°C. The protein concentration was tested using a BCA kit. Equal amounts of protein samples (40 µg/well) were separated electrophoretically on 10% SDS-PAGE and transferred to PVDF membranes. After blocking in PBS-Tween 20 containing 5% skimmed milk (diluted with 0.1% PBST) for 1 h, the membranes were each incubated with a primary antibody against legumain (1:1,000; cat. no. ab125286), N-cadherin (1:1,000; cat. no. 8457, Cell Signaling Technology, Inc., Danvers, MA, USA), and GAPDH (1:1,000; cat. no. 5174, Cell Signaling Technology, Inc.) overnight at 4°C, washed three times with PBS-Tween 20, and incubated with a goat-anti-rabbit peroxidase-labeled secondary antibody for 1 h. After three washes with PBST, the membrane was incubated with SuperSignal West Pico and then exposed.


**Informed consent:** Informed consent has been obtained from all individuals included in this study.
**Ethical approval:** The research related to human use has been complied with all the relevant national regulations, institutional policies, and in accordance with the tenets of the Helsinki Declaration, and has been approved by the Ethics Committee of Xinhua Hospital Affiliated to Shanghai Jiao Tong University School of Medicine Approval No. XHEC-D-2015-103, 28 February, 2015.

### 2.3 Legumain gene knockdown by siRNA or overexpression by plasmid transfection

SiRNA was transfected to reduce legumain expression in NB cell lines SH-SY5Y and IMR32. Cells were seeded in a 6-well plate, grown to the confluence of 60–70%, and transfected with RNAimax 9 µL (Thermo Fisher Scientific, Inc.) mixed with siRNA 90 pmol (Genomeditech, SHH, CHN) for each well. Proteins in the cell lysate were extracted 72 h after transfection and used in the following experiments. Carboxyfluorescein and western blot analysis were used for assessing knockdown efficiency.

Plasmids carrying human legumain genes were transfected to increase legumain expression in the NB cell line SK-N-BE2. Cells were seeded in 6-well plates, grown to a confluence of 60–70%, and transfected with Lipo2000 5 µL (Thermo Fisher Scientific, Inc.) mixed with legumain containing 2.5 µg plasmid (GeneChem, SHH, CHN) in each well. Proteins in the cell lysate were extracted 72 h after transfection and used in the following experiments. The efficiency of transfection was examined by western blot analysis.

### 2.4 Invasiveness and migration assay

The invasiveness and migration ability of the different experimental groups below were tested by the scratch test or transwell test. NB cells (SH-SY5Y and IMR32) were seeded in a 6-well plate and grown to a confluence of 60–70%. After 48 h of legumain gene knockdown by siRNA, a line created by a 20 µL pipette tip was scratched into a confluent monolayer of cells to investigate cell motility. Pictures were taken immediately after the scratch and 24 h later, and distance measurements were carried out three times.

After legumain gene knockdown by siRNA or legumain gene overexpression by plasmid transfection for 48 h, cells that had invaded a polycarbonate membrane or Matrigel-coated polycarbonate membrane were fixed in 10% paraformaldehyde for 30 min, stained with crystal violet for 10 min, and then were counted under a microscope. Active mature recombinant human legumain (AEP, 2 µg) or legumain inhibitor (AEPI, 5 µL, 20 mmol/L) were added to the cells seeded in the transwell chambers, and pictures were taken 24 h after treatment.

### 2.5 Effects of recombinant human legumain (RhLegumain) and legumain inhibitors on EMT

RhLegumain purified protein (Novoprotein, SHH, CHN) and legumain inhibitor (AEPI, 5 µL, 20 mmol/L) were added into NB cell line SH-SY5Y to test their invasiveness and migration. RhLegumain (5 µg, 1 mg/mL) was activated under activation solution (50 mM citric acid, 121 mM Na_2_HPO_4_, 1 mM EDTA, and pH 4.1) for 3 h before adding to the cells [24], and legumain inhibitor (diluted in DMSO) was incubated with the culture medium for 30 min before adding to the cells, the hydrolyzing effect of AEP or enzyme-inhibition by AEPI was checked by hydrolyzing the substrate Z-ala-ala-asn-AMC (GL Biochem, SHH, CHN). The vehicle was used as a control. Transwell test and EMT markers were also examined.

### 2.6 Statistical analysis

All experiments were repeated at least three times. The results are expressed as the mean value ± SEM. Student’s paired *t*-test was used to analyze the differences between the two groups. A *p*-value <0.05 was defined to indicate statistical significance.

## 3 Results

### 3.1 Legumain expression in NB

**Figure 1:**
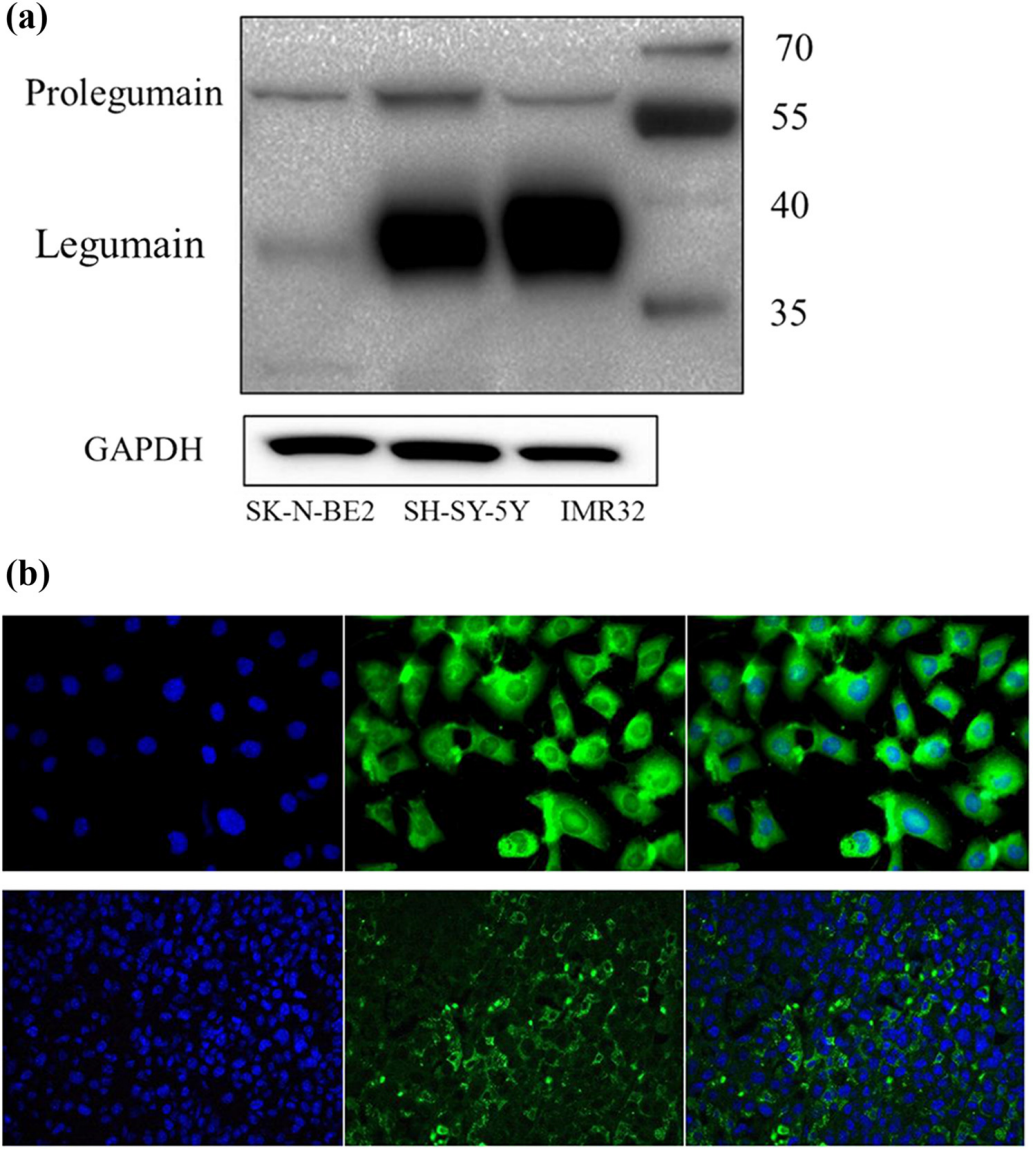
Legumain expression in NB. (a) Legumain expression in NB cell lines SK-N-BE2, SH-SY5Y, and IMR32 by western blotting analysis. (b) Immunofluorescence staining in SH-SY5Y (20×) and tumor xenograft (10×). Legumain (green fluorescence) was localized in the cytoplasm and extracellular matrix. Cell nuclei are stained blue.

Legumain expression in SK-N-BE2, SH-SY5Y, and IMR-32 cells was confirmed by western blotting (Figure 1a). SK-N-BE2 had more precursor forms than the active mature forms of legumain, but the other two cell lines, SH-SY5Y and IMR-32, had more active forms than precursor forms. In the immunofluorescence assay, legumain expression was mainly localized to the cytoplasm of the tumor cells. Besides, examining tumor xenograft models from SH-SY5Y also confirmed high levels of legumain, mainly in the cytoplasm and extracellular matrix of NB (Figure 1b).

### 3.2 N-Cadherin was positively correlated with active legumain expression in NB tumor samples

**Figure 2:**
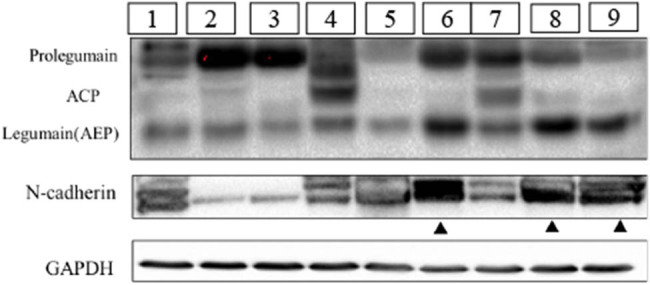
Correlation analysis of Legumain and N-cadherin in the clinical NB samples in the western blot assay. Three forms of legumain can be tested in NB, including precursor form (prolegumain, 56 kDa), intermediate form (ACP, 46 kDa), and mature active form (legumain, 36 kDa). Tumor samples were labeled from number 1 to number 9. Samples as the triangle pointed (numbers 6, 8, and 9) presented with a high level of active mature legumain (36 kDa) and N-cadherin. Samples pointed by the arrow (numbers 2 and 3) showed less expression of legumain and N-cadherin.

Nine NB tumor samples were collected from nine patients at the Shanghai Xinhua Hospital. Clinical pathology confirmed the diagnosis of NB. Legumain was detected in all nine samples. Three forms of legumain were observed in the tumor samples and were identified as the precursor form (prolegumain, 56 kDa), intermediate form (ACP, 46 kDa), and mature active form (AEP, 36 kDa). As observed in our experiment, when the active form of legumain (36 kDa) levels were relatively high (sample numbers 6, 8, and 9 shown by a triangle), the corresponding N-cadherin expression was also at a relatively high level. On the contrary, while mature active legumain expression was low but prolegumain was high, N-cadherin expression was relatively low (sample numbers 2 and 3, shown by the arrow). Therefore, active mature legumain is positively correlated with N-cadherin expression, which suggests that active legumain may regulate N-cadherin expression (Figure 2).

### 3.3 Legumain increases invasiveness and migration by modulating EMT

**Figure 3:**
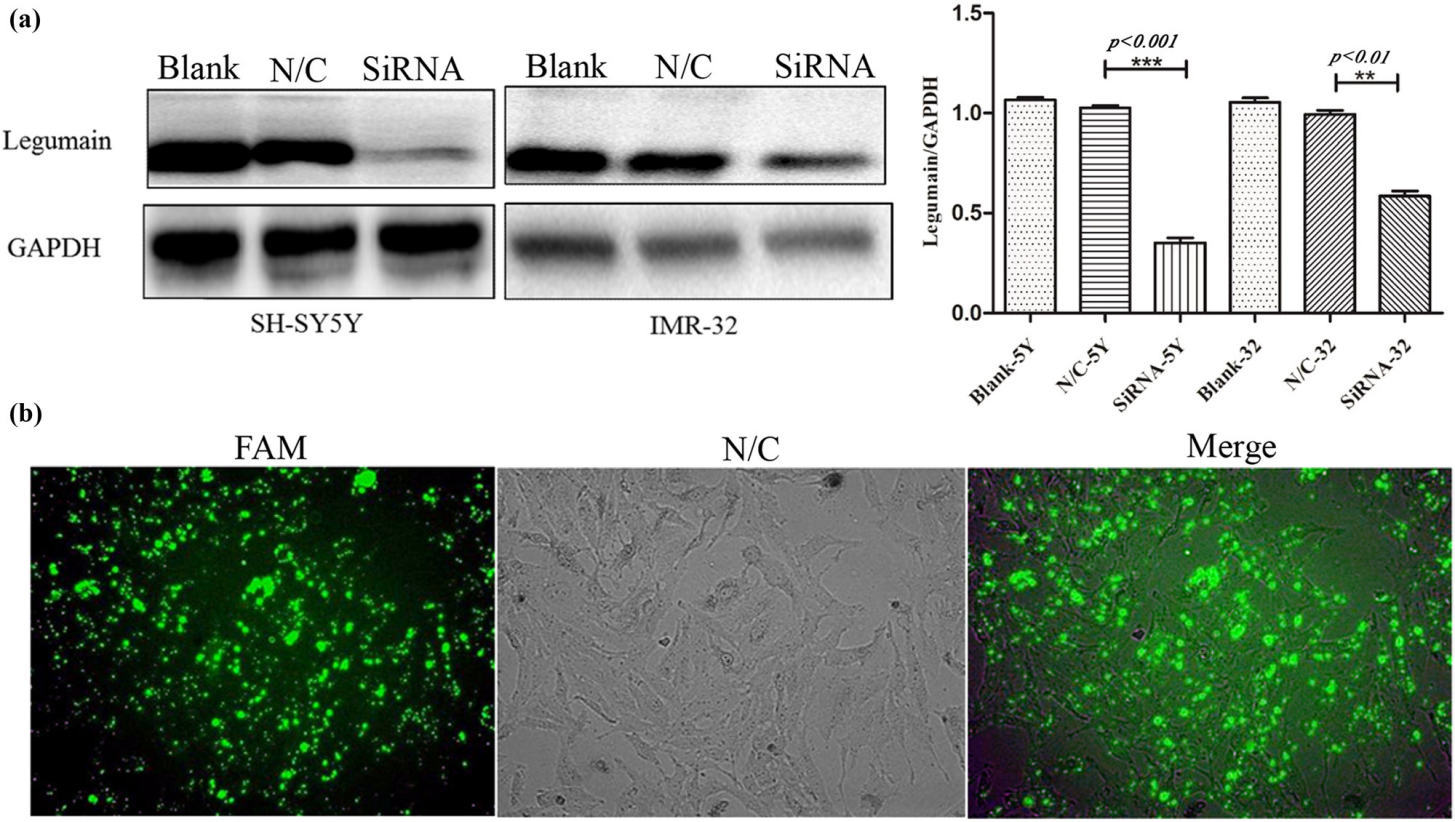
Knockdown of legumain by siRNA. (a) Knockdown of legumain by siRNA in SH-SY5Y and IMR32 cell lines was confirmed by western blot analysis. (b) Knockdown efficiency was also confirmed by FAM fluorescence coloration.

**Figure 4:**
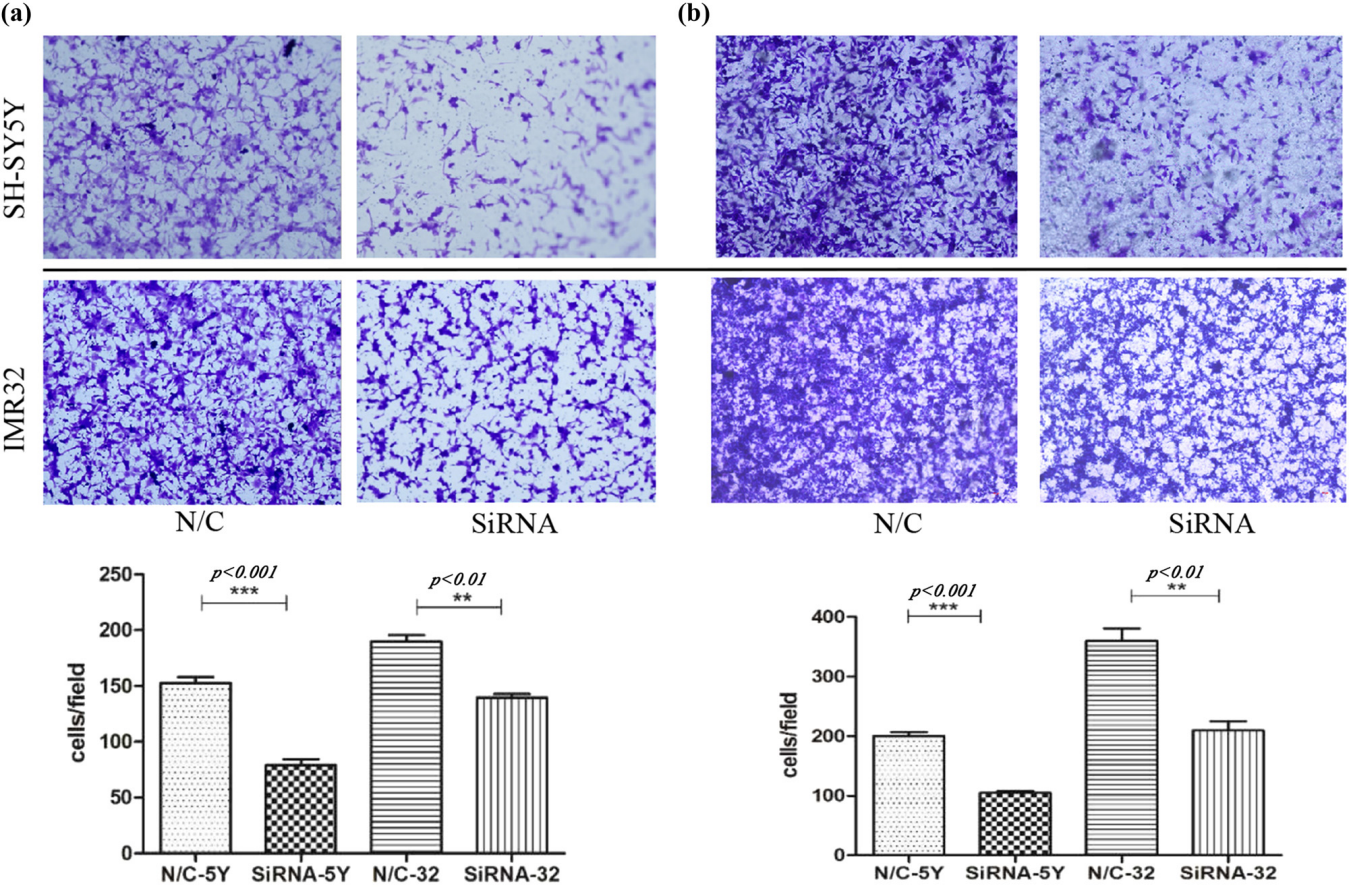
Migration and invasion tests by transwell assay. Cell counts (10×) passing through the transwell chamber after a legumain knockdown by siRNA decreased in cell lines SH-SY5Y and IMR32 compared with the control, *p <* 0.05 (***p < 0.001 and **p < 0.01). The procedure and statistic analysis is similar. (a) and (b) is different only in treatment of transwell membrane. In order to give brief explanation, a and b can be briefly summarized.

**Figure 5:**
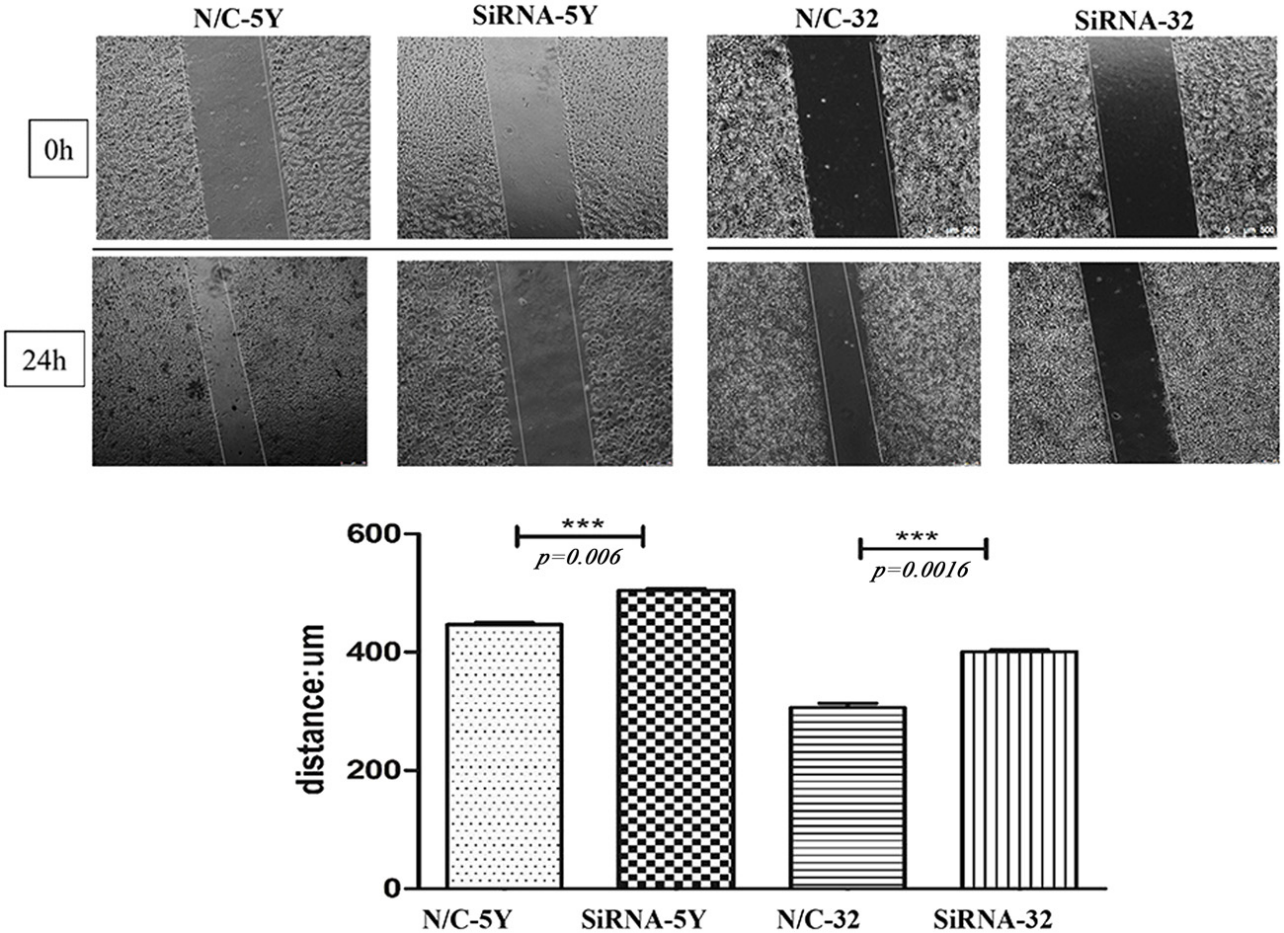
Gap distance (10×) increased after legumain knockdown by siRNA in SH-SY5Y and IMR32 in scratch assay compared with the control, *p* < 0.05 (***p < 0.001 and **p < 0.01).

Western blot analysis showed a diversity of legumain expression in the three NB cell lines, a low level in SK-N-BE2 and a high level in SH-SY5Y and IMR32. Therefore, legumain was knocked down by siRNA in the cell lines SH-SY5Y and IMR32 (Figure 3a). Knockdown of legumain reduced the migration and invasion of SH-SY5Y and IMR32, which was examined by a transwell assay (Figure 4) and a scratch assay (*p* = 0.006 for SH-SY5Y; *p* = 0.0016 for IMR32) (Figure 5).

**Figure 6:**
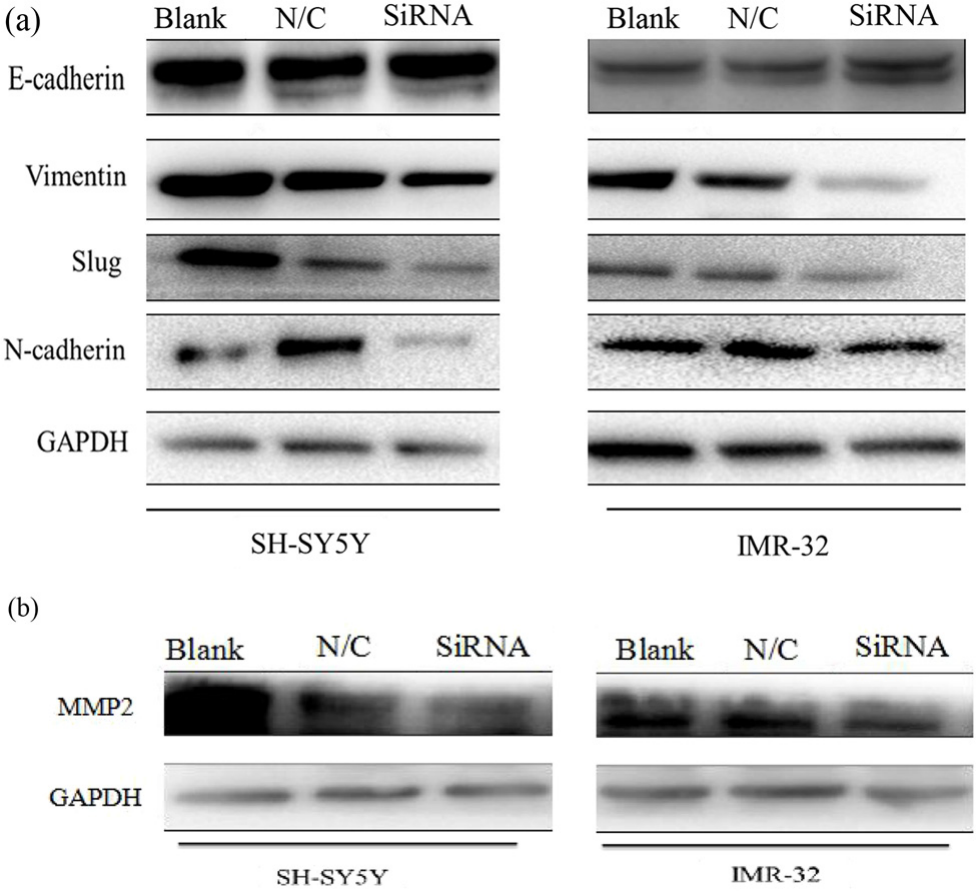
Changes in EMT markers after knockdown of the legumain gene. (a) Mesenchymal markers including vimentin, slug, and N-cadherin decreased in cell lines SH-SY5Y and IMR32 after knockdown of legumain in western blot analysis, but epithelial markers like E-cadherin increased accordingly. (b) MMP2 also decreased after the knockdown of legumain.

After legumain knockdown or overexpression for 72 h, EMT markers were examined (Figure 6), such as E-cadherin (an epithelial marker), N-cadherin, slug, and vimentin (mesenchymal markers). After siRNA knockdown, MMP2 was also examined to evaluate the knockdown efficiency. All mesenchymal markers examined were downregulated after legumain knockdown, but epithelial markers were upregulated.

**Figure 7:**
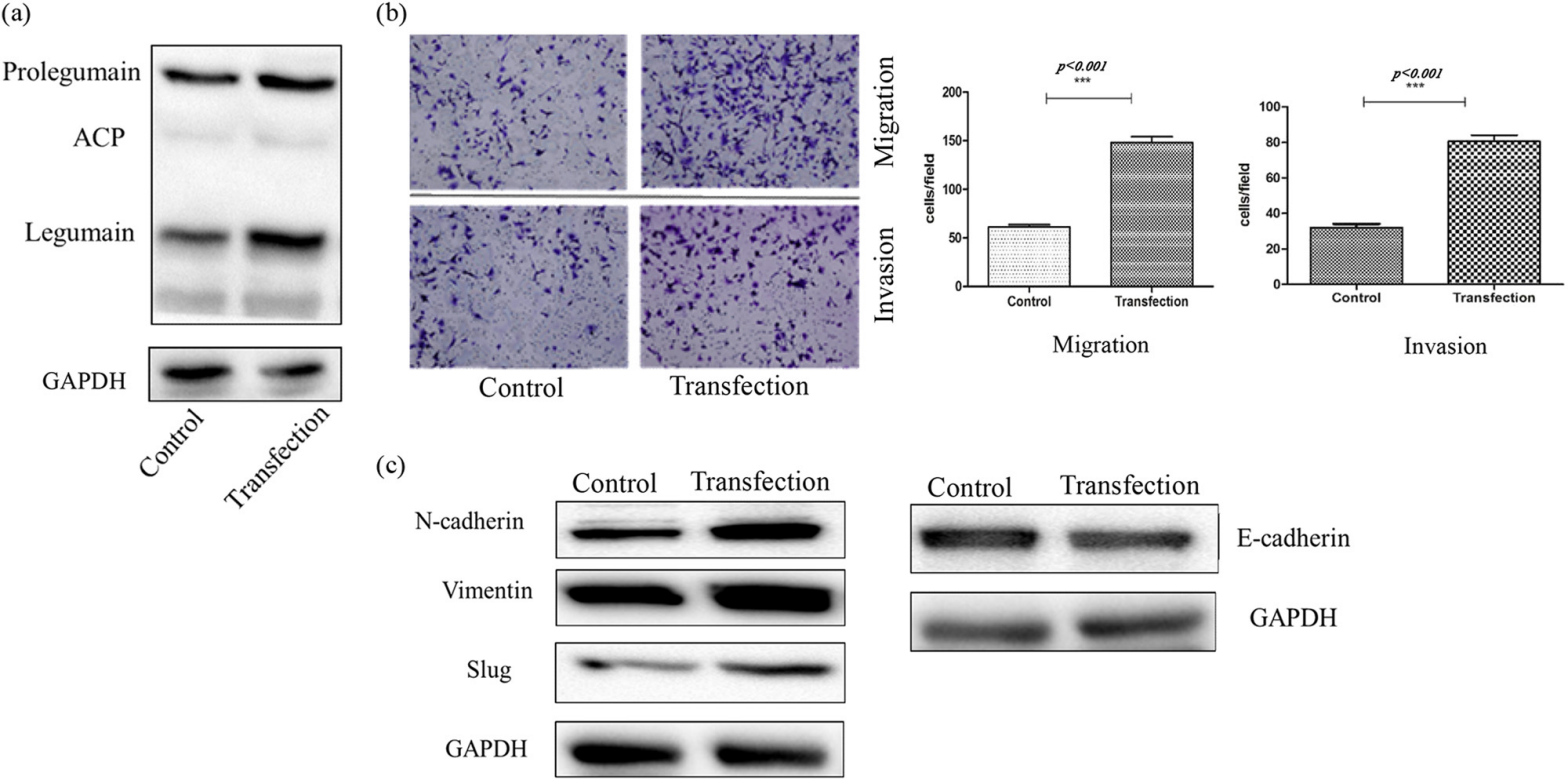
(a) Overexpression of legumain by a plasmid carrying legumain coding gene transfection in cell SK-N-BE2 was proved by western blot. (b) Migration and invasion ability increased in transwell assay after legumain overexpression by plasmid transfection, *p <* 0.05. (c) Mesenchymal markers above increased after legumain overexpression, but epithelial marker decreased.

Legumain expression was amplified by plasmid transfection into the cell line SK-N-BE2 (Figure 7a), so the effect of legumain on migration and invasion was explored. Transfection of a plasmid carrying the legumain gene increased the migration and invasion of SK-N-BE2 cells (Figure 7b) in a transwell assay. When legumain was overexpressed, mesenchymal markers like N-cadherin, vimentin, and slug were upregulated significantly, while epithelial markers E-cadherin was downregulated slightly (Figure 7c).

### 3.4 Effect of legumain on EMT relies on its AEP activity and can be inhibited by AEPI

**Figure 8:**
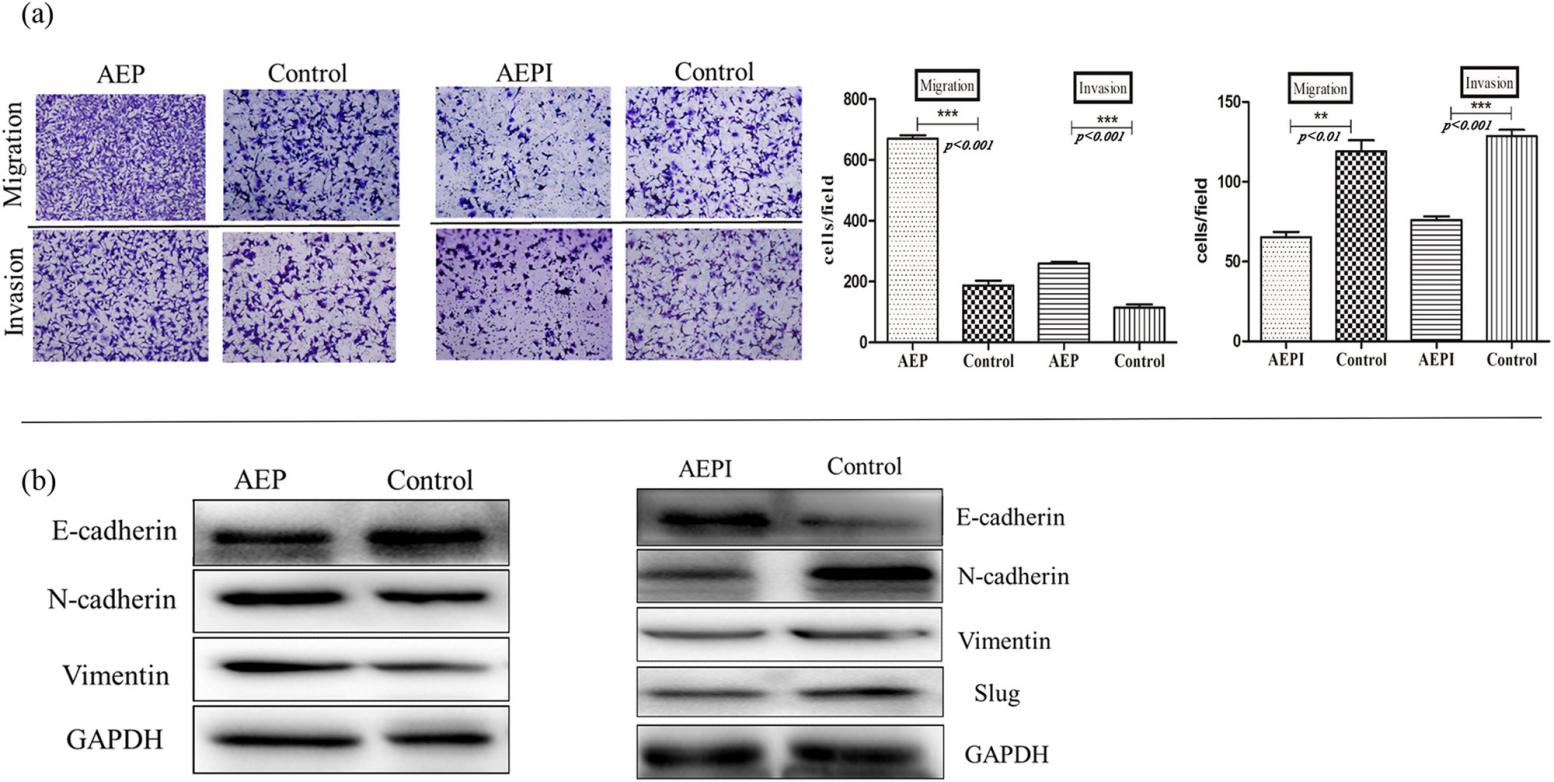
Changes in EMT markers after AEP or AEPI treatment. (a) Cell counts passing through the transwell chambers in transwell assay with or without Matrigel increased after AEP treatment in SH-SY5Y, but AEPI worked contrarily (10×), *p <* 0.05. (b) Mesenchymal markers increased after AEP treatment in SH-SY5Y in western blot assay, but epithelial markers decreased; mature active legumain (AEP) worked contrarily in the changes in EMT markers.

RhLegumain and the AEPI on EMT were introduced to examine AEP activity during EMT of NB cells. RhLegumain was activated into AEP in pH 4.1 activation buffer, and the effect of AEP and AEPI was confirmed by hydrolyzing the substrate Z-Ala-Ala-Asn-AMC. 24 h after activation of AEP or applying AEPI, migration and invasion abilities changed in SH-SY5Y cells (Figure 8a), with an enhancement of the migration and invasion ability by AEP, but inhibition of these by AEPI. Meanwhile, EMT markers examined (Figure 8b) with the above mesenchymal markers were upregulated by AEP and downregulated by AEPI. The epithelial marker E-cadherin was downregulated after treatment with AEP but upregulated after treatment with AEPI.

## 4 Discussion

The cysteine protease legumain (prolegumain) can develop into an AEP and an ACP in some aberrant situations [25,26]. Consistent with other reports on legumain in tumors [27], legumain was also found to be expressed in NB, where it is mainly distributed in the cytoplasm and extracellular matrix. There was a disparity in relative amounts of the 56 kDa prolegumain and 36 kDa active mature legumain in the three cell lines SK-N-BE2, SH-SY5Y, and IMR32, representing the inactive zymogen and mature active form, respectively. Expression of AEP is increased under the conditions of starvation, acidic pH, hypoxia, and other stress situations, so we used a relatively neutral culture medium and the same culture conditions (37°C, 5% CO_2_) to avoid factors like starvation, acid pH, and hypoxia so that different levels of mature active legumain could be correlated with different levels of legumain inhibitors-cystatins. Cystatin C and cystatin E/M are the most potent legumain inhibitors. Cystatins may regulate the activity of AEP through substrate competition for the active site [28]. The use of cystatin C to inhibit EMT and morphological transformation stimulated by transforming growth factor-beta was reported [29].

Wallin et al. [30] reported cystatin C in NBs. More cystatin C is present in the NB cell line SK-N-BE2 than in SH-SY5Y, consistent with our finding that less active mature legumain was present in SK-N-BE2 than in SH-SY5Y. Therefore, a disparity in cystatin C may explain the difference in levels of mature 36 kDa legumain between these cell lines. The IMR32 cell line is the most malignant type-I form of NB [31], and the abundant active legumain in IMR32 may also support legumain as a marker of tumor malignancy.

EMT has been shown to be important in regulating tumor metastasis and played a central role in tumor development [32,33]. A recent report also found that mesenchymal markers were correlated with a poor prognosis of NB, and mesenchymal markers should be regarded as potential markers for NB prognostication [34]. Slug is an important factor in NB because it is involved in gastrulation, development, and migration of neuronal precursors during embryonic development, and it also plays a role in tumor metastasis. Because NB is an embryonic neuroblastic tumor, slug may be involved in its development.

Vimentin is a typical marker of EMT [35]. Overexpression of vimentin correlates with accelerated tumor growth, invasion, and a poor prognosis in some cancers [36,37]. Vimentin’s overexpression during metastasis [38] suggests its central role as a metastasis promoter. In the regulation of EMT by legumain, either through gene overexpression of legumain or by extrinsic active mature legumain treatment, slug and vimentin were both upregulated and when legumain was inhibited by AEPI, slug and vimentin were downregulated, suggesting a major role of legumain in EMT of NB.

Lammens et al. [39] reported that all NB samples express N-cadherin and might be a valid target for treatment. Our experiments showed the expression of active mature legumain (AEP) and N-cadherin were positively correlated well in NB. Gene knockdown or plasmid transfection of legumain was carried out to verify the effect of legumain on EMT and N-cadherin expression, besides AEP and AEPI, were also introduced to elucidate the AEP activity of legumain changes in N-cadherin. Both gene knockdown and AEPI were able to downregulate N-cadherin expression in our *in vitro* experiments, and there was an upregulation of N-cadherin after legumain plasmid transfection and AEP treatment.

Legumain’s functions in digestion, antigen processing, and signaling via processing/activation are mainly attributed to its AEP activity [40]. Previous reports in adult solid tumors indicated a positive correlation between legumain and tumor malignancies, and our research proved legumain could regulate EMT through its AEP activity. The multi-branched and context-dependent activation process of legumain illustrates that proteases can act as signal transducers and decision-makers. Still, there is much we do not know. It is unclear how it triggers EMT as a transcriptional factor or just an interaction regulator between matrix proteins in the tumor stroma. Additional study of legumain in the future is essential.
